# Make it complicated: a qualitative study utilizing a complexity framework to explain improvement in health care

**DOI:** 10.1186/s12913-019-4705-x

**Published:** 2019-11-14

**Authors:** Marie Höjriis Storkholm, Pamela Mazzocato, Carl Savage

**Affiliations:** 1 Department of Learning, Informatics, Management and Ethics, Medical Management Centre, Karolinska Institutet, Tomtebodavägen 18A, 171 77 Stockholm, Sweden; 2 Department of Obstetrics and Gynecology, Aarhus University Hospital, Aarhus, Denmark

**Keywords:** Quality improvement, Complexity science, Process mapping, Change management, Obstetrics and gynecology

## Abstract

**Background:**

Successful application of Quality Improvement (QI) methods is challenging, and awareness of the role context plays has increased. Complexity science has been advocated as a way to inform change efforts. However, empirical support is scarce, and it is still difficult to grasp the practical implications for QI interventions. The aim of this study was to use a complexity-based leadership framework to explain how managers in a clinical department addressed external requirements to cut costs without compromising patient outcomes and experience.

**Methods:**

Explanatory case study design of a Danish OB/GYN department tasked to improve efficiency. Data came from documents, 30 interviews, and 250 h of observations over 3 years. A Complexity Analysis Framework that combined two complexity-based leadership frameworks was developed to analyze all changes implemented to reduce cost, while maintaining clinical quality.

**Results:**

Managers reframed the efficiency requirement as an opportunity for quality improvement. Multiple simple, complicated, and complex situations were addressed with an adaptive approach to quality improvement. Changes were made to clinical pathways for individual conditions (*n* = 37), multiple conditions (*n* = 7), and at the organizational level (*n* = 9). At the organizational level, changes addressed referral practice, physical space in the department, flow and capacity, discharge speed, and managerial support. Managers shared responsibility with staff; together they took a “professional path” and systematically analyzed each clinical pathway through process mapping, attentive to patterns that emerged, before deciding on the next steps, such as a engaging in a complex process of probing – the iterative development and testing of new responses.

**Conclusions:**

Quality improvement efforts could benefit from an understanding of the importance of learning and sharing responsibility to deal with the co-existing degrees of contextual complexity in modern health care. By “making things complicated” through a systematic analysis that engages staff in an open and reflective dialog, clinical praxis and established organizational structures can be questioned and improved. The Complexity Analysis Framework could then help managers to identify improvement opportunities, know when to implement technical solutions, and when to keep abreast of emerging patterns and allow appropriate responses to complex challenges to evolve.

## Background

Quality improvement (QI), the systematic application of methods and strategies to change provider behavior and the organization, [1] has been proposed as a way to improve quality and thereby reduce costs [2, 3]. These concurrent goals to improve population health, patient experience, and reduce cost have recently been framed as the Triple Aim [4].

Although QI includes a wide range of similar methodologies related to cycles of improvement, tools and techniques, organizational leadership support and commitment, and staff involvement, [5] organizations often struggle to achieve the desired results [6–8]. Despite the promise of a linear approach, in practice, change efforts are often experienced as chaotic, full of unexpected events, discontinuous activities, and shifting goals where the dominant discourse does not match organizational realities [9–13]. The source of this mismatch could be that QI is incorrectly applied, [10] or that it does not fit with the context of application.

Context plays a large role in change efforts, [14–17] where the same intervention in terms of content and process can yield different results in different settings [17–20]. In the Profound Knowledge of Improvement, context largely relates to knowledge about systems. Since the health care context is often described as complex, [21] and QI efforts often as well, advances in our understanding of complexity could potentially help deepen our understanding of how we can improve the success of quality improvement efforts in health care.

### Complexity science in health care

Complexity science offers a dynamic view of organizational reality relevant to change management in health care [8, 20, 22]. Complexity science is not a single theory, but a study of living systems that has matured in different scientific fields [22]. With a complexity lens, organizational change can be understood as a non-linear and unpredictable process with elements of co-evolution, self-organizing, and emergence balanced on the edge of chaos [23–25]. The last two decades have seen an increase of publications about complexity theory in health care, [26] e.g. to explain system, innovation, and implementation failures or guide educational development [8, 25, 27–29]. However, the hoped for paradigm shift has not materialized [30]. It is difficult to grasp the practical implications of using complexity theory to improve care quality without frameworks which translate the underlying logic of complexity into actionable behaviors. And more empirical studies are needed to explore the effect and possible benefit of complexity-informed improvement efforts [31].

Studies in health care have shown that the effectiveness of the same QI approach may vary dependent on the complexity of the situation being addressed [32, 33]. Several authors have proposed that by identifying the different levels of complexity in the challenges we are facing, we may become better at developing appropriate responses [27, 29, 34]. It is also possible to discern a shift in how complexity is conceptualized, from a focus on the number of nodes/agents in a system to complexity as a process of interactions and responses and their subsequent transformative effect [35]. Thus, managers, could through their interactions with staff, have an important role to play when an organization embarks on a quality improvement effort in a complex environment.

One such leadership approach that acknowledges different levels of complexity, Adaptive Leadership, describes how to diagnose and act according to three types of “situational complexity” [36]. Heifetz, who uses many examples drawn from medicine, suggests that responses to these situations can be categorized into technical (simple problems), technical and adaptive (complicated problems), or adaptive (complex challenges). Each level has specific characteristics related to the problem definition, solutions, and the kind of work needed to develop appropriate responses. Thus, simple and complicated problems have clear problem definitions, can be solved by managers alone or together with staff, and the solutions will be clear or require learning. Complex (adaptive) challenges often require learning to define the issue, and to develop a response. The more complex the challenge, the more the locus of responsibility shifts to the staff [36, 37].

Another leadership framework that acknowledges similar levels of complexity is Cynefin [38]. It adds decision processes suitable for different contextual situations, including chaos, as well as diagnostic support for managers to determine which level of complexity is present. In health care, the Cynefin framework has been used to explore health promotion efforts and to explore “the black box” of quality improvement [39, 40].

These two leadership frameworks, grounded in complexity science, both suggest that situations should be addressed in relation to their level of complexity, i.e. simple problems may benefit from applying the organization’s current know-how following usual decision processes, while complex problems are often resistant and require that individuals and the organization question their way of working to allow improvements to emerge [36, 38].

In this study, we combined these complexity-based leadership frameworks to explain how managers in a clinical department addressed external requirements to cut costs without compromising patient outcomes and experience.

## Methods

### Study design

An explanatory case study design [41] with multiple data sources to study a real world pursuit of the Triple Aim in the Obstetrics and Gynecology (OB/GYN) department at Aarhus University Hospital (AUH) in Denmark.

### Study setting

AUH has an annual budget of ≈€870 million, 990 beds, and employs 10,000. Since 2009, AUH is transforming into a “super hospital” tasked with providing both general and highly specialized care [42]. The 10-year process involves merger and relocation, stipulated by an 8% efficiency requirement. In January 2013, GYN had eliminated five beds through unit mergers and implemented minimal invasive surgery. In May, the OB/GYN department was asked to further reduce beds by 36%, and the staff and the ≈€26.8 million budget by 10%. At the time, the department had 102,024 outpatient visits (71,623 OB) and 8443 admissions (5874 OB) and 423 employees. Department management consisted of an obstetrician, a midwife, and a nurse. Based on analyses for internal monitoring and improvement purposes of data from national quality registries and patient satisfaction surveys, and which will be presented in a separate paper, the department was able to do so without evidence of compromised quality or patient experience.

### Data collection

Between October 2013 and January 2017, 198 documents (working documents, process maps, presentations, meeting notes, action-plans, hospital reports, newspaper articles, and departmental newsletters), 30 semi-structured interviews, hospital administrative systems data, and observations (> 250 h over 3 years) conducted in camps, workshops, and meetings were collected by the first author.

For the interviews, 18 staff members were randomly selected within purposively chosen personnel groups (nurses, doctors, midwives, and medical secretaries). All 12 clinical managers in the department were interviewed. The interview guide covered the three essential ingredients of change (context, process, and content) [17] and the hour-long open-ended interviews were digitally recorded. The same interviews were used in a previous study, where we explored how staff and managers understood the change imperative inherent to the Triple Aim and the mental models underlying their understanding [43]. The interview guide can be found in its entirety as part of the first author’s PhD thesis [44].

### Data analysis based on the combined Cynefin and adaptive leadership frameworks

Interviews were transcribed *verbatim* by the first author and read through to develop familiarity. To build the case description, a qualitative content analysis [45] of the interviews and observational field notes was conducted to describe the change process.

Observational field notes and 86 documents that specifically included information about the implemented changes, were organized chronologically and coded in NVivo (Version 10). From the documents and field notes, 1100 codes were extracted and categorized into those related to changes in clinical pathways or the organizational level and further subdivided into obstetrics and gynecology. Organizational changes were grouped thematically. Thus, each clinical pathway or organizational improvement project often consisted of several changes. To move beyond an analysis of barriers to change, we focused on implemented changes to understand *what actually works*. The first author’s knowledge of clinical praxis and previously employed at the department prior to the changes was used to determine which changes were realized. Follow-up interviews with department managers were used to validate this analysis. Coding and the subsequent analyses were done in English to involve all the authors to strengthen trustworthiness and mitigate the risk for bias [46], particularly as the first author had previously been employed at the department.

Data from observations, documents, and interviews were triangulated to create abstracted descriptions for each improvement project.

We combined the Adaptive Leadership and the Cynefin frameworks by situational level of complexity. This created a more comprehensive framework (Table 1) that differentiates between simple, complicated, complex, and chaotic situations and links them to managerial actions. The levels of contextual complexity relates to: 1. The problem definition (clear or requires learning), 2. the response (clear or requires learning), 3. the primary locus of responsibility for the work (manager, manager and staff, and staff > manager), 4. the kind of work (technical, technical and adaptive, and adaptive), and 5. the decision-making process, where *categorize*, *analyze*, *probe,* and *act* were key verbs unique to each level of complexity. We field tested the framework with approximately 50 managers in health care executive training programs and found that it resonated well with participants, who frequently described that their solutions were often more technical than adaptive.

**Table 1:** Complexity Analysis Framework for QI in Health Care

Situation	Problem definition	Response	Primary locus of responsibility for the work	Kind of work	Decision-making process
Simple	Clear *Ordered universe with clear causality*	Clear *Answers are self-evident, undisputed, and can be determined based on facts and evidence*	Manager	Technical *Often a question of solution implementation*	Sense Categorize Respond *Responses are developed through the categorization of existing facts*
Complicated	Clear *Ordered universe with clear causality, though not perceived by everyone*	Requires learning *May contain multiple correct answers; involves analysis, expert consultations, and the creation of working groups. Requires coordination and collaboration, time consuming, often with a tradeoff between the “best” answer and making a decision, but complete data becomes available … eventually*	Manager and staff	Technical and adaptive *Often a question of solution implementation and evolution of new responses through experimentation and discovery*	Sense Analyze Respond *Responses are developed based on analyzing several options*
Complex	Requires learning *Unordered universe without clear causality*	Requires learning *No right answers exist – decisions often based on incomplete data*	Staff > manager	Adaptive *Often a question of evolution of new responses through experimentation and discovery*	Probe Sense Respond *Responses are developed by letting them emerge by testing different ideas*
Chaotic	Requires action *to create stability in an unordered universe*	Requires action *to stabilize in order to gain perspective and enable diagnosis – no point to search for right answers*	Manager	Technical	Act Sense Respond *By first acting, order can be established so that a response can be developed*

Based on the analysis framework, the type of situation, problem definition, response, and decision process for each improvement project were categorized by all authors together (Additional file 1). By asking if the problem definition and response were clear or required learning, we sought to corroborate our diagnosis of the type of situation. Categorization of the decision-making process involved identification of the key verbs in the process summaries for each quality improvement project.

## Results

### Case description

The Danish health system redesign to create 16 super hospitals generated external pressure to improve efficiency. This triggered an extensive change process:

I do not think we had developed it [the change strategy and process] if we had not gotten this task. It may well be that we had developed some small things, but this whole big “set-up” is initiated, it is driven by the requirements we must meet. (Department manager 1)

Department management quickly understood that the efficiency requirement was beyond typical cost reduction approaches. To handle their initial internal turmoil, they took time to reflect and realized that they needed to work in close collaboration with staff:

Completely perplexed, I spent a weekend in almost total despair, because I could not figure out anything. What would be wise? Which way should we go? I went for a long walk on Sunday, and suddenly I thought, “OK, this is what we need to do: we need to work intensively with it [the efficiency requirement], and with the patient pathways, and we have to take a professional path!” It's the only thing that works; all that managerial "You have to understand, we have to..." is not something the employees have any use for. (Department manager 2)

**Fig. 1:**
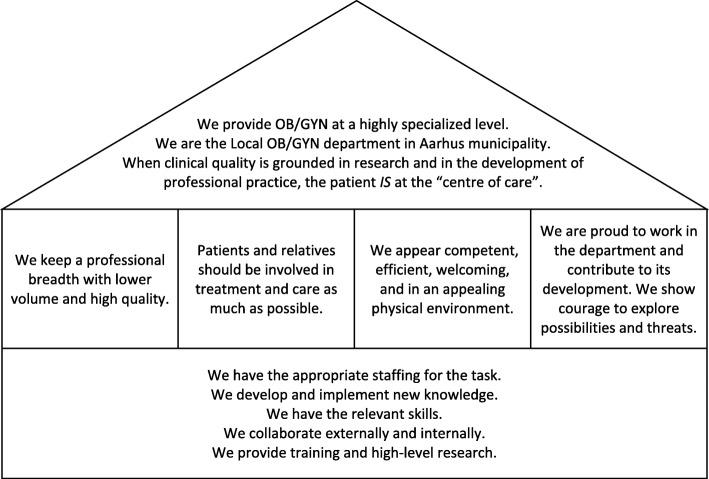
The aspirations, principles, and foundational prerequisites (in descending order) of the department

The 11 months preceding closure of the first beds in February 2014 commenced with a four-month process where department managers developed a “master plan” with first-line managers. It was refined together with the department staff committee and nurses responsible for gynecological research and education. The purpose was to outline the department’s core principles, strategies, and vision in response to the efficiency requirements. The vision was summarized in a figure that captured the aspirations, principles, and foundational prerequisites of the department (Fig. 1). The plan was presented to the whole department. Further small-group discussions and feedback from each section followed after which it was accepted as the way forward.

Included in the plan was a description of the “professional path” – the idea that the change process should be anchored in the inter-professional desire of staff to improve patient experience and outcomes within the constraints of reduced physical floor space and staff: [43].

The purpose of setting up a number of working groups that work with patient pathways and the organization is to stay focused on the professional task, which we must solve in our department with the same high quality in the new hospital, but adapted to the new [smaller] physical space and with reduced staff availability (Master plan, September 2013).

Managers continually re-emphasized that the purpose was to improve care, not just cut costs. Interdisciplinary working groups were appointed and given a clear mandate to review and generate ideas for more efficient clinical pathways. Working group chairs were introduced to lean methodology and process mapping by an external consultant. In order for managers to be able to focus on one section at a time, and to optimize cross-sectional learning, department managers arranged 24-h off-site kick-off camps for obstetrics and then gynecology. Inspired by the Amabile et al. article, “Creativity under the gun,” managers introduced the spartan camps as *“*being on a mission*”*, shielded from external disturbance, where the task was meaningful, challenging, and needed to be solved together [47]. Working groups began by mapping all inpatient pathways, starting with high-volume conditions or those perceived to be resource heavy by the department managers, who sensed that the pathway could be improved. Department managers supported each working group chair and together they facilitated the process with questions based on four principles described in the master plan:

1. Patients prefer to be at home
2. Coordination and collaboration within each pathway and across organizational boundaries can be improved
3. Hospital-based care should be reserved for those with serious conditions or need highly specialized care
4. Functions should be combined across organizational boundaries and competencies utilized across teams.

Managers introduced pathway mapping as a process of asking questions about what works well, is problematic, and ideas to address both. They encouraged staff to continually ask, “Is this really needed? What do our patients prefer? Is there evidence for this?”

Following the initial camps, obstetrics held five more off-site camps, whereas gynecology held shorter on-site meetings and workshops. Department managers continued to facilitate these without involving external consultants. Collaboration and knowledge-sharing between working groups was encouraged, especially in the camps, and involvement of external stakeholders was expected. An iterative, back-and-forth process between the working groups, who generated ideas, and department managers, who prioritized which ideas should be developed and explored, emerged. Design thinking and prototyping were introduced and recommended for idea development and testing [48]. Throughout, ideas were posted on bulletin boards and presented by first-line managers at staff meetings and the regular plenary meetings. A blog (obstetrics) and videos were added to the internal webpage and newsletters were distributed weekly.

### Multiple changes implemented

Fifty-three improvement projects were identified (Additional file 1). Changes were made to address situations in clinical pathways for individual conditions (*n* = 37; 27 OB and 10 GYN), multiple conditions (*n* = 7; 1 OB and 6 GYN), and at the organizational level (*n* = 9; 4 OB, 4 GYN, and 1 both). The first and second of the four principles were the impetus for most of the changes. Organizational situations addressed were: referrals, physical space, flow and capacity, discharge speed, and managerial support (Additional file 2).

Between February–October 2014, 11/40 (OB) and 10/30 (GYN) beds were closed stepwise, with a further two beds in January 2017. The remaining two were relocated to the emergency department when it opened in 2018. From July 2014, changes were continually made in clinical pathways. Guidelines were developed to manage possible overcrowding and bed occupancy rates were monitored closely and evaluated during morning conferences.

### Case analysis

When managers realized the path forward was unpredictable and uncertain, they reframed the efficiency requirement as an adaptive challenge that they needed to learn more about in order to develop an effective response. The efficiency requirement to reduce beds, staff, and budget could have been interpreted by department managers as a simple problem with a clear solution involving technical work to implement changes. In such a scenario, responsibility would reside with department management to make the decisions and then inform staff in a way that would generate acceptance and buy-in. Instead, department managers realized, after some reflection, that the task required a high level of collaboration with staff. Therefore, they began by defining an overarching strategy together with staff, by first reaching agreement around the aspirations, principles, and the foundational pre-requisites for the department. With this base, they chose a “professional path”, which required staff to make use of their medical competences to better define the problems and develop possible responses to improve clinical pathways, i.e. to strive for the Triple Aim.

### Complexity framework analysis

In Table 2, we present the analysis of the case with illustrative examples, more details can be found in Additional file 1.

**Table 2:** Categorization of changes implemented in individual clinical pathways and at the organizational level with illustrative examples

Situation (*n* = 53)	Problem definition^a^	Response^a^	Primary locus of responsibility for the work	Kind of work	Decision-making process^a^
Simple 18 (34%)	Clear 38 (72%) *Unnecessary visits by pregnant patients with suspected intrahepatic cholestasis because patients were seen more acutely than evidence suggests is warranted.*	Clear 6 (11%) *To use recently developed regional guidelines to reduce unnecessary admissions due to trauma in pregnancy.*	Manager 3 (6%) *Managers were responsible for introducing the new guideline on “trauma in pregnancy” in the electronic guideline collection.*	Technical 3 (6%) *To implement the new guideline on “Trauma in pregnancy”.*	Sense Categorize Respond 2 (4%)
Complicated 20 (38%)		Requires learning 47 (89%) *Developing networked, cross-sectional collaborations with interprofessional teams able to design individualized treatment plans for newborns who had lost weight.*	Manager and staff 13 (24%) *Redesigning the physical space of the new obstetrics clinic by merging four units. Managers held the responsibility for changing the physical space and organizational structures and staff was responsible for developing and testing the new workflows and new patient pathways*.	Technical and adaptive 32 (60%) *Reduce admissions due to postpartum hemorrhage. The technical work involved raising the limit for how much bleeding could be accepted without having to admit the patient for further observation. This change was based on the realization that the previous limits were based on insufficient evidence. The adaptive work involve changing the “better safe than sorry” culture.*	Sense Analyze Respond 4 (8%)
Complex 15 (28%)	Requires learning 15 (28%) *How to redesign the physical space of the new obstetrics clinic that arose from the merger of four units to improve patient flow and coordination and collaboration across organizational and professional silos.*		Staff > manager 37 (70%) Responsibility for development of the group B streptococcal (GBS) test to prevent unnecessary administration of IV-antibiotics and the subsequent unnecessary admission for observation was held by a senior physician.	Adaptive 18 (34%) Iterative approach to redesign the physical space and the flow of patients and staff in the obstetrical unit.	Probe Sense Respond 0 (0%)

^a^The problem definition and responses could not be differentiated for simple and complicated, and complicated and complex situations, respectively. The majority of the decision-making processes did not fit the theoretically pre-defined patterns as they involved more steps and are therefore not categorized in the table

### Situation, problem definition, and response

The distribution between simple, complicated, and complex situations was 34, 38, and 28% respectively, with no chaotic situations identified (Table 2). Most problem definitions (70%) were clear, however, only a few responses (11%) were clear from the start. Most of the 53 responses implemented required learning (89%) to develop.

### Primary locus of responsibility

Managers worked with staff on most situations by facilitating open dialog at the camps around the improvement of pathways. Managers reviewed the ideas that had been generated and selected which ones to pursue. They then returned responsibility to the staff working groups for the development and implementation of the selected ideas (70%). When they shared responsibility with staff as equal partners (24%) it was in those situations that required changes of organizational structures or physical space. In three projects (6%), managers easily identified a solution that they directed be implemented.

### Kind of work

In only three improvement projects (6%) was the kind of work technical. Most work involved a combination of technical and adaptive work (60%) where the problem might be clear but the response was not readily so. Primarily adaptive work (34%) occurred when learning was required to identify the challenge and develop an appropriate response.

### Decision-making process

The decision-making process matched the process outlined in the framework in six improvement projects (12%), i.e. *categorization* was only used as the point of departure in two simple and *analysis* in four complicated situations. Instead most decision processes began with an analysis (92%), i.e. process mapping. The subsequent response could then lead to a categorization or probing or a combination thereof. One example was the development of the group B streptococcal (GBS) test to prevent unnecessary administration of IV-antibiotics and the subsequent unnecessary admission for observation. The decision process began with analysis through process mapping. Then the response was to conduct a validation study to validate the GBS-test in the Danish population [49]. Different possible set-ups for analyzing the test were probed in the labor ward and the laboratory, and the response was to implement the best solution.

### Analysis of the complexity level alignment

Reading Table 2 horizontally, it becomes clear that managers’ decision processes did not match the levels of situational complexity. Instead, they began with the complicated process of analysis. Initially, they *sensed* that there was a possibility for improvement. This guided their change strategy of extensive *analysis* in interdisciplinary camps, which made it possible to *probe* for new responses. The systematic and collective process mapping of all clinical pathways made it possible to address the differing degrees of complexity that coexisted in the same organizational context. From a managerial perspective, the systematic analysis together with staff became the start of a larger probing process about how to most effectively pursue the Triple Aim. It also became clear to staff and managers that certain ideas for improvement could have multiple effects outside the particular pathway or address several different challenges across the organization. Thus, “turning every stone” allowed complex patterns to emerge.

## Discussion

The combination of two complexity-based leadership frameworks helped us identify, in this real-world case study, that the task of cutting costs without compromising patient outcomes and experience requires that managers address multiple levels of complexity, i.e. simple, complicated, and complex situations. What worked for the managers we studied was to start with a complicated analysis approach, which created an opportunity to better understand and respond to the situation. We identified three patterns related to how managers approached and led the pursuit of the Triple Aim – reframing, continual engagement, and turning every stone.

### Reframe efficiency demands as a mandate to improve quality and care experience

Attempting to address complex problems can provoke anxiety. Shifting the locus of responsibility to interprofessional communities united to improve care could help lessen this. However, it requires that managers invest in developing facilitation skills that enable them to link the intellectual capabilities of people and capitalize on the interactive dynamics and collective learning of the group such that responses will emerge [50, 51]. The managers in this study did just that – they applied what could be considered a “generative leadership” strategy that enabled staff and managers to learn as they progressed [52]. This approach fits well with the complex situation they were facing [36]. Previously, we found that the “professional path” strategy, as outlined in the master plan, resonated well with staff mental models of change in health care [43]. In the context of the constraints formed by the task to improve care quality and experience in the shadow of the demand to reduce costs. i.e. the “Triple Aim” [4], the system was able to self-regulate and allow appropriate responses to emerge [38]. In this case, the “Triple Aim” acted as a generative image, [53] guiding managers and staff through conversations about improving clinical pathways, not cost cutting. In their actions, the managers appear to have understood that, “the key to success is working with, rather than trying to simplify or control, complexity” [54].

### Continually engage staff through reflective dialogue and shared responsibility

Managers continually encouraged honest and open dialogue in their interactions with staff – key to effective leadership in complex situations as they help generate a diversity of ideas for further probing [38, 55, 56]. Open dialogue can contribute to organizational development [57] by creating a safe environment where ideas are allowed to fail [58, 59]. Managers reinforced this by actively participating in the dialogue not only as facilitators, but also as learners. They maintained perspective by moving between active participation on the “dance floor” and reflections on the process from “the balcony” to develop next steps and actions plans [60].

In the interdisciplinary process mapping and iterative decision-making processes, managers were able to fully immerse themselves in the discussions. They facilitated deeper reflection through their questions [35, 61]. These questions were built on shared principles, and helped staff realize and reassess assumptions to develop new organizational narratives [57, 62, 63]. Challenging these assumptions is vital to improve health care [64–66]. Manager’s willingness to question all steps of all pathways enabled staff and managers to rethink established narratives, [57] such as “better safe than sorry” thinking, which can lead to unnecessary defensive medical practices. Staff and managers developed shared mental models when managers created interfaces for knowledge sharing and leveraging boundaries [67]. Thus, the facilitated process mapping became a learning opportunity [68].

### Turn every stone and make things complicated to reveal complex challenges

Process mapping is a common QI technique, but in this case, the blanket approach of extensive analysis made it possible to realize when solutions were obvious or when staff and managers needed to learn more to understand problems and develop appropriate responses. While iteration is another basic principle in QI, it is seldom done in practice [10, 32]. When QI is used without iteration, there is a risk for quick and short-term fixes – i.e. technical solutions for adaptive challenges [36]. This could explain the critique of QI as tools and techniques for simple problems, but not complex challenges [32, 33, 69]. This case also calls into question a tendency among health care organizations to reduce quality improvement approaches to superficial and top-down implementation of technical solutions, i.e. tools and methods [69]. A complicated decision process, while it may sound daunting, could in fact provide an opportunity to better define the situation and generate awareness of when it is appropriate to take the time to reframe, engage staff, shift the locus of responsibility, and together learn how to improve.

To navigate complexity in a “simpler” way, the Complexity Analysis Framework could help professionals and health care managers more ably adapt QI efforts to develop appropriate responses suitable for modern health systems. We found poor empirical support for the decision-making processes in the analysis framework. The implication of our findings for practitioners is that contextual complexity varies within the same setting and the decision-making process should vary as well – but *after* an initial analysis to help understand and “diagnose” the degree of complexity. Then, the decision can be made whether to implement technical solutions or learn how to develop more appropriate responses and how much responsibility to shoulder. And the decision process will most likely reflect a much more “messy” reality.

### Limitations

Conducting research on one’s own organization has an inherent risk of bias. We attempted to mitigate this through triangulation, cross-referencing and validation with department managers, journaling, and continual reflection among co-authors without connections to the department. Gynecology had fewer working groups and camps, which generated less data, which may explain why fewer changes were identified in this section. While we worked to include many different views in the interviews, we could have expanded the validation to include other staff to contribute to an even more nuanced understanding of the implementation. The thick description of contextual situations, decision processes, and responses is provided to improve translation to other contexts.

The complexity analysis framework was used to analyze an ongoing improvement effort and its ability to inform prospective strategy development has yet to be tested. We recognize the challenges inherent to diagnosing the level of contextual and situational complexity, which is why the “analysis first” decision process may prove beneficial to such a diagnostic process. The framework and conclusions could be further tested by analyzing failed QI efforts.

## Conclusion

This case illustrates that it may be possible for improvement projects to become more successful if the quality improvement approach combines QI methods with an understanding of the importance for learning and shared responsibility to deal with co-existing degrees of contextual complexity in modern health care. External demands to improve efficiency can be reframed as a mandate to improve professional practice. By making it complicated, managers can invite staff to engage in open and reflective dialog and systematically question the way work is done in order to address both technical problems and adaptive challenges. Managers would therefore do well to err on the side of learning over implementation when pressed to choose. This would help managers keep abreast of the emerging patterns as they develop, encourage established narratives to be questioned, and possible responses to evolve.

## Supplementary information

**Additional file 1.** Analysis of the obstetrics and gynecology clinical pathways and organizational changes.

**Additional file 2.** Table of organizational situations addressed.

## Data Availability

The datasets generated in the analysis that support the findings of this study are included in this published article and its supplementary information files, entitled “Additional file 1” and “Additional file 2”. The interview and observational datasets generated and/or analyzed during the current study are not publicly available in the interest of maintaining confidentiality and anonymity as per agreement with study participants during data collection. Illustrative quotations have been provided to support the analysis. We are willing to discuss the data upon reasonable request directed towards the corresponding author.

## Abbreviations

AUH: Aarhus University Hospital
GYN: Gynecology
OB: Obstetrics
OB/GYN: Obstetrics and Gynecology
QI: Quality Improvement
