# Supplementary material for: Make it complicated: a qualitative study utilizing a complexity framework to explain improvement in health care
Source: BMC Health Serv Res. 2019 Nov 14;19:842. doi: 10.1186/s12913-019-4705-x (PMC6857274; doi:10.1186/s12913-019-4705-x)
Supplement: Supplementary file 1 — Additional file 1. Analysis of the obstetrics and gynecology clinical pathways and organizational changes. [file 12913_2019_4705_MOESM1_ESM.docx]

# Additional File 1

## Abbreviations:

OB: Obstetrics

GYN: Gynecology

OB-org: Obstetrical section – Organizational level

GYN-org: Gynecological section – Organizational level

AUH: Aarhus University Hospital

PH: Patient Hotel

ABC: After Birth Clinic

MC: Midwife Clinic

GP: General Practitioner

OOC: Obstetrical Outpatient Clinic

GOC: Gynecological Outpatient Clinic

EOC: Emergency Obstetrical Clinic

EGC: Emergency Gynecological Clinic

FMU: Fetal Medicine Unit

DSU: Day Surgical Unit

PW: Pregnancy Ward

OB/GYN: Obstetrics and Gynecology

OR: Operating Room

CTG: Cardiotocography

MRI: Magnetic resonance imaging

Ped. Department: Pediatric Department.

Abd. Surgery: Abdominal Surgery

GA: General Anesthesia

GI-function: Gastro Intestinal function

KAD: Kateter à demeur

GBS: Group B Streptococcus

FAQ: Frequently Asked Questions

OHSS: Ovarian Hyperstimulation Syndrome

LOS: Length of Stay

BP: blood pressure

PE: Preeclampsia

# ANALYSIS

## Obstetrics

| **NUMBER** | **ID NUMBER** | **DESCRIPTION/SUMMARY OF ANALYSIS** | **CONTEXTUAL**  **SITUATION** | **PROBLEM DEFINITION** | **RESPONSE** | **PRIMARY LOCUS OF RESPONSIBILITY FOR THE WORK** | **KIND OF WORK** | **DECISION-MAKING**  **(WORK) PROCESS** |
| --- | --- | --- | --- | --- | --- | --- | --- | --- |
| **1** | **1 OB**  Trauma in pregnancy | Simple. Clear problem definition – Avoid unnecessary admissions. Analysis of the pathway did not lead to new responses as the group decided to adopt a technical solution, i.e. adhere to a new regional guideline that categorized trauma into high or low risk, with only high risk needing a 24-hour observation period., which matched the result of the analysis. | Simple | Clear | Clear | ? Managers | Technical | **Analyze**  Respond  **Categorize**  Respond |
| **2** | **2 OB**  Post partum hemorrhage | Simple. Clear problem definition – Avoid unnecessary admissions. Clear causality in the literature – analysis with no supporting evidence for admission at a certain safety limit. This challenged the “better safe that sorry” cultural attitude among staff. Changed safety limits for admission in the department guidelines based on a new categorization of the amount of bleeding that should lead to admission based on literature. | Simple | Clear | Requires learning (Complicated) | Staff supported by managers | Adaptive and then technical | **Analyze**  Respond  **Categorize**  Respond |
| **3** | **3 OB**  Lactation after birth | Simple, clear problem definition. – Avoid unnecessary admissions by more flexibility in labor ward (stay a few more hours) and collaboration with ABC. Multiparous with previous successful breastfeeding could discharge directly from labor ward – other could be care for in the PH with support from ABC, thus avoid admission to the maternity ward. | Simple | Clear | Requires learning | Staff supported by managers | Adaptive and then technical | **Analyze**  Respond  **Analyze**  **Probe** (walking nurse/ABC) |
| **4** | **4 OB**  Gemelli  (Non-identical) | Simple. Clear problem definition – too many outpatient visits. Development of response and work process were complicated as a mapping (analysis) of all the visits was made and which, when analyzed, revealed that double-visits were not needed. These were then removed.  Before this analysis, a specialist gemelli midwife performing group-consultations had begun in the MC – that further made a reduction in visits possible. | Simple | Clear | Required learning (trough cross sectional analysis) | Staff supported by managers | Adaptive then technical | **Analyze**  Respond **Categorize** Respond |
| **5** | **5 OB**  Fetal mal-formation | Simple – Clear problem definition – too many unnecessary outpatient visits in AUH. Redistribution of activities to regional departments based on clear problem definition, simple context, clear solution, technical work based on categorization of diagnostic work related to need for specialist competence. Furthermore, a categorization of patients/fetus with heart disease was conducted so that fetal medical specialist examined that only patient that needed specialist ultrasound. | Simple | Clear | Required learning | Staff supported by managers | Adaptive and then technical | **Analyze**  Respond  **Categorize** Respond |
| **6** | **6 OB**  Anal Sphincter injury | Simple situation with a clear problem definition. Unnecessary admissions, without supporting evidence. The solution was technical but informed by literature review and experiences from other departments and involved more collaboration between ABC, physiotherapy and labor-ward. | Simple | Clear | **Clear** – only because the analysis had already been conducted before the camps | (Staff supported) by managers | Technical  (Required collaboration and coordination) –the analysis had already been conducted. = Adaptive | **Categorize**  Respond |
| **7** | **7 OB**  Children in pediatric care | Simple situations with a clear problem, unnecessary admission of mother – the response included an expanded collaboration with neo and PH – so that children in pediatric care and waiting for diagnostic test could be admitted in pediatric department or PH – Maternity follow up can be done in the ABC instead of during admission in the maternity ward. Tested a “solution” using a walking nurse. | Simple | Clear | Required learning | Staff supported by managers | Adaptive and then technical | **Analyze**  Respond  **Categorize** **Probe** |
| **8** | **8 OB**  Children with infection | Simple situation with a clear problem definition (logistics) –expanded collaboration with PH – ABC –  Antibiotics- treatment in PH – cared for by “walking nurse” and parents. | Simple | Clear | Required learning | Staff supported by managers | Adaptive and then technical | **Analyze**  Respond  **Probe** |
| **9** | **9 OB**  Prurigo /intrahepatic cholestasis (ICP) | The problem was defined as clear and simple. The context was simple. The response, while it required some coordination, could have been simple since it did not take a long time to develop, however it did require some coordination of activities. The rewriting of referral guidelines was quick, but the review of the new guidelines and the spread of the information took a longer amount of time. The patient group was categorized as not being necessary to be seen acutely, which guided subsequent response development, however, this was arrived at after a pathway analysis, which suggests that a complicated work process was used to solve a simple problem. Staff and middle managers shared the locus of responsibility developed the solution together without much discussion department management. | Simple | Clear | Clear | Staff supported by managers | Technical and adaptive | **Analyze**  Respond **Categorize** Respond |
| **10** | **10 OB**  Neonatal  Weight loss | Simple situation with a clear problem definition (to many unnecessary admissions) – the response was developed through a prototyping process. And included a collaboration between neonatologist, maternity nurse and ABC, that made individual plans – not based on 10% cut off but an individual evaluation of the baby and family. | Simple | Clear | Required learning | Staff supported by managers | Adaptive | **Analyze**  Respond  **Probe**  **Analyze**  Sense  Respond |
| **11** | **11 OB**  Breech presentation | Simple situation with a clear problem definition – the MC could not diagnose if a baby was a breech presentation because they had no ultrasound and training. The response was to get have ultrasound available in the MC and train a midwife coordinator to scan breech or no-breech. | Simple | Clear | Required learning | Staff supported by management | Adaptive and technical | **Analyze**  Respond  **Probe**  Respond |
| **12** | **12 OB**  Children with jaundice | Simple situation with a clear problem definition – the care of children with jaundice could only be handled in the maternity ward due to lack of nursing competences in other facilities.  The pathway was analyzed and the light mattress was tested in the PH and finally implemented in home care also, allowing babies to receive light treatment for jaundice at home or in PH and be followed by ABC-nurse. | Simple | Clear | Required learning | Staff supported by management | Adaptive then technical | **Analyze**  Respond  **Probe**  Respond |
| **13** | **13 OB**  Premature  Children | Simple situation with a clear problem definition  Premature heating mattress at home allows premature to go home earlier, this new approach was tested in collaboration with the pediatric department. | Simple | Clear | Required learning | Staff supported by management | Adaptive and technical | **Analyze**  Respond  **Probe**  Sense  Respond |
| **14** | **14 OB**  Diabetes in pregnancy | A complicated situation with several aspects (i.e. better safe than sorry, correct and changed risk stratification) and a clear problem definition – avoid unnecessary admissions. Clear causality, though not everyone can see it, guidelines were not followed because of uncertainty and worry of staff – better safe than sorry (defensive medicine). The response was to review the literature and the categorize patients. Guidelines were changed with new risk stratification and an emphasis on that staff follow department guidelines when admitting patients. | Complicated | Clear | Required learning | Staff supported by management | Adaptive and technical | **Analyze**  Respond  **Categorize**  Respond |
| **15** | **15 OB**  Bleeding in Pregnancy | A complicated situation with several aspects (i.e. better safe than sorry, correct diagnosis before admission). A working group analyzed several care pathways and identified a clear problem of too many unnecessary admissions. They responded by reviewing the literature and based on that improved the diagnostic process before admission so that staff felt able to address the tendency for admissions to be “better safe than sorry” i.e. defensive medicine. Observation reduced from 48 to 24 hours after fresh bleeding | Complicated | Clear | Required learning | Staff supported by management | Adaptive and technical | **Analyze**  Respond  **Categorize**  Respond |
| **16** | **16 OB**  Preeclampsia (mild) | Complicated situation with a clear problem definition. Too many unnecessary visits of preeclampsia patients in the EOC.  The response was to develop a “solutions shop” in the MC using BP monitoring and to change guidelines for follow-up in pregnancy clinic or to hand over the follow up process to a GP or the MC. In the case of induction – monitoring in PH is possible.  The response involved that the visitation guideline was changed – high BP should be verified by coordination midwife before referral and follow-up of mild PE can be done in midwife clinic or by GP –when referred to OOC then patient are seen by a OB/GYN resident | Complicated | Clear | Required learning | Staff supported by managers | Adaptive and Technical | **Analyze**  Respond  **Categorize**  **Probe**  Sense  Respond |
| **17** | **17 OB**  Vacuum assisted birth | Complicated situation with a clear problem definition – a technical approach was used to analyze patient data and reject a simple solution (re-categorization of all patients) and learn about causality (analysis of admission data) and therefore adopt a response that required each midwife to tailor an individual plan for the patient (baby). This required increased flexibility in labor ward and collaboration with ABC, PH, and maternity ward. | Complicated | Clear | Require learning | Staff supported by managers | Adaptive | **Analyze**  (Pathway and admission-data)  Respond  **Analyze**  **Probe**  Sense  Respond |
| **18** | **18 OB**  Neonatal hypoglycemia | Complicated situation a clear problem definition, that required improved collaboration and coordination to develop a response – To avoid unnecessary admissions by improving flexibility in labor ward (stay a few hours more) and collaboration with maternity ward, ABC and PH | Complicated | Clear | Required learning | Staff supported by managers | Adaptive and technical | **Analyze**  Respond  **Analyze**  **Probe**  Sense  Respond |
| **19** | **19 OB**  Vulnerable pregnant | Complicated situation with a clear problem definition, the current approach does not take in to account the varying need of patients and therefore a more flexible approach was needed to avoid unnecessary admissions. Individual plans instead of a fixed 5 days observation period – home visit team to conduct network meetings that reduces waiting time under admission. Possible to stay in PH and parent wing of Ped. Department also. | Complicated | Clear | Required learning | Staff supported by managers | Adaptive | **Analyze**  Respond  **Probe**  Sense  Respond |
| **20** | **20 OB**  Cesarean section CS | Complicated situation with a clear problem definition, too long length of stay. The pathway was analyzed and changes were made in pain-medication, improving recovery of GI function and guidelines for discharge. Involving anesthesiologist –and that prophylactic antibiotics could be given in PH | Complicated | Clear | Required learning | Staff supported by managers | Adaptive | **Analyze**  Respond  **Probe**  Sense  Respond |
| **21** | **21 OB**  Urine retention after birth | Complicated problem definition, it appeared clear at first but through analysis and discussions between nurses and midwives, the clarity of the problem changed to be rather prober pain treatment, instead of unnecessary admission of patients with KAD.  If the patient really had urine retention she could be admitted to PH or go home with KAD with support from ABC – change admission guidelines after complicated birth. | Complicated | Clear  Wasn’t clear for everyone | Required learning | Staff supported by managers | Adaptive | **Analyze** Respond  **Probe**  Sense  Respond |
| **23** | **23 OB multiple**  Fetal Growth Retardation | Complicated situation with a clear problem definition, too many unnecessary ultrasounds. The response was to update the categorization template so it was adjusted to the national guideline for Fetal Growth Retardation. | Complicated | Clear | Required learning | Staff and  Management | Adaptive and  Technical | **Analyze**  **Categorize** Respond |
| **24** | **24 OB**  Medical induction | Complex situation with a problem definition that requires learning, i.e. too many admissions for medical induction, too long waits, and too many patients waiting in the EOC, all this prolonged treatment and disturbed patient flows. This was analyzed, and it became clear that improving medical induction was a complex situation that required further learning. Different improvement suggestions were developed and then probed, induction initiated in different locations of the department, with a faster medical regime or at different time in the day. Which lead to many changes being adopted. | Complex | Requires learning | Required learning | Staff supported by managers | Adaptive | **Analyze**  Respond  **Probe**  Sense  Respond |
| **25** | **25 OB**  Normal birth | Complex situation with a problem definition that required learning. After analysis of the process maps for individual medical conditions, staff and managers realized that many patients in the EOC did not present with an emergency but “just” labor or came for induction of birth. The solution was to develop a pathway for patients in latent phase to be examined in the labor ward (Implementation of partus telephone) instead of EOC – this led to a more streamlined process for patient in “normal birth”. | Complex | Requires learning | Required learning | Staff supported by managers | Adaptive and technical | **Analyze**  Respond  **Categorize**  **Probe**  Sense  Respond |
| **26** | **26 OB**  Preterm premature rupture of membranes  PPROM | Complex situation with a problem definition that requires learning based on awareness that there was no evidence that admittance generates better outcomes. The response was developed through an iterative innovation process that including research and external partners, in testing and implementing home monitoring (CTG, blood pressure, temperature) –this process was led by a department senior doctor/professor, in a project about Tele-health – which he was part time employed in. | Complex | Requires learning | Required learning | Staff supported by managers | Adaptive | **Analyze**  Respond  **Probe**  Sense  Respond |
| **27** | **27 OB**  Preeclampsia – (Previous severe or in current pregnancy)  (Tele-Health) | Complex situation with a problem definition that requires learning based on awareness that there is no evidence that admittance generates better outcomes. The response was developed through an iterative innovation process including research and external partners, in testing and implementing home monitoring (CTG, blood pressure, temperature) | Complex | Requires learning | Required learning | Staff supported by managers | Adaptive | **Analyze**  Respond  **Probe**  Sense  Respond |
| **28** | **28 OB**  Short cervix | Complex situation with a problem definition that requires learning based on awareness that there is no evidence that admittance generates better outcomes. The response was developed through an iterative innovation process including research and external partners, in testing and implementing home monitoring (CTG, blood pressure, temperature) | Complex | Required learning | Required learning | Staff supported by managers | Adaptive | **Analyze**  Respond  **Probe**  Sense  Respond |
|  | **ID NUMBER** | **DESCRIPTION/SUMMARY OF ANALYSIS** | **CONTEXTUAL**  **SITUATION** | **PROBLEM DEFINITION** | **IMPROVMENT** | **PRIMARY LOCUS OF RESPONSIBILITY FOR THE WORK** | **KIND OF WORK** | **DECISION**  **(WORK) PROCESS** |
| **29** | **1 OB org**  **Referral external/ internal**  Redefined and expanded role for nurse coordinator in OOC  (Gatekeeper)  Midwife coordinator in MC  (Gatekeeper)  Partus telephone | Complex situation with a problem definition that required learning; for example, how to expand the role for nurse coordinator was unclear. The final response was to make that organizational role in to an overall gatekeeper for all acute/sub-acute referrals that covered several medical conditions.  Midwife: Complex situation with a problem definition that required learning about the reasons behind too many unnecessary referrals from MC to EOC, and FMU. This was solved through an analysis of many outpatient pathways and the establishment of a midwife coordinator function in the MC (gatekeeper). A “partus telephone” was established, which allow laboring woman to contact the labor ward directly and on be referred through the EOC. | Complex | Requires learning | Required learning | Staff supported by  managers | Adaptive | **Analyze**  many pathways (outpatient care)  Respond  **Probe**  Sense  Respond |
| **30** | **2 OB org**  Change if the physical space of EOC | Complex situation with a problem definition that required learning – The problem was identified through the analysis of all the obstetrical pathways, too much transportation, inflexible staffing, waiting time, and too much activity in the EOC. The responses were developed in an iterative process of adaptive learning to improve the flow in the obstetrical department by dividing patient guided by their need for emergency vs. elective care, before or after birth ect. Furthermore, the new units that was created were relocated next to each other, which allowed for task shifting and task specialization and increased collaboration. These changes involved: Establish new obstetrical settings with 4 units (ABC, EOC, PW and labor-ward) in the same physical space. | Complex | Required learning | Required learning | Staff and managers | Adaptive | **Analyze** all pathways,  Respond  **Probe**  Sense  Respond |
| **31** | **3 OB org**  Flow and capacity  Changed staffing | Complex situation with a problem definition and a response that required learning. The response was developed through analysis of many pathways, combined with physical space-changes and a requirement to reduce nursing staff. It included task shifting of staff in the pregnancy ward from nurses to midwives; establishment of collaboration between midwifes and nurses in ABC, maternity ward and PH. A closer collaboration had a synergistic effect on competency levels – staff (nurses and midwifes) developed better knowledge and understanding about each other’s competencies. | Complex | Required learning | Required learning | Staff and managers | Adaptive | **Analyze**  Respond  **Probe**  Sense  Respond |
| **32** | **4 OB org**  Faster discharge of obstetrical patient | Complex situation with a problem definition that required learning. The response was developed by reviewing multiple pathways and involving collaborators in other sectors, the ABC and PH. It included more formalized agreement of transfer off patients, early discharge with follow up in ABC and telephone hotline, children that could be treated in PH, accelerated discharge after CS, shorter observation for bleeding in pregnancy, individualized plans for vulnerable pregnant and home-network team and a culture change aiming to be able to discharge 24/7  A “walking nurse” that handles PH-patients was included in this change | Complex | Required learning | Required learning | Staff and managers | Adaptive | **Analyze**  Respond  **Probe**  Sense  Respond |

## Gynecology

| **Number** | **ID NUMBER** | **DESCRIPTION/SUMMARY OF ANALYSIS** | **CONTEXTUAL**  **SITUATION** | **PROBLEM DEFINITION** | **IMPROVEMENT** | **PRIMARY LOCUS OF RESPONSIBILITY FOR THE WORK** | **KIND OF WORK** | **DECISION**  **(WORK) PROCESS** |
| --- | --- | --- | --- | --- | --- | --- | --- | --- |
| **33** | **1 GYN**  Fertility FAQ | Simple situation with a clear problem definition: Too many unnecessary telephone calls. The solution was developed through process mapping. And included expanding information on webpage with a FAQ. | Simple | Clear | Clear | Staff | Technical | **Analyze**  Sense  Respond |
| **34** | **2 GYN**  Ascites drainage | Simple problem with a clear problem definition – too much waiting time for the procedure = unnecessary admission time.  The response was developed through process mapping of several pathways (ovarian cancer, palliation and OHSS), which was developed through probing and knowledge sharing in teaching sessions.  The response included that ascites drainage could be performed in the ward instead of OR and by all Gynecological specialists. | Simple | Clear | Required learning | Staff | Adaptive and technical | **Analyze**  Respond  **Probe**  Sense  Respond |
| **35** | **3 GYN**  Cervical dysplasia | Complicated situation with a clear problem definition. Too many patients did not show up, got some double-info if they went to surgery, surgery was performed in GA, and too many very admitted after surgery. The response included changed visitation, longer opening hours, preoperative info via telephone, aligning info with DSU, use local anesthesia as when doing surgery instead of GA. Handling postoperative bleeding in EGC instead of OR. A gynecological-oncology specialist provides pathology answers via telephone. | Complicated | Clear | Required learning | Staff supported by managers | Adaptive and technical | **Analyze**  Respond |
| **36** | **4 GYN**  Ovarian cancer | Complicated situation with a clear problem definition: too long admission and inability to reach the aim of the national cancer bundles. The response was developed through process mapping, a categorization that there was a need for highly specialized care, and establishing a closer collaboration with other department including new visitation guide, and faster mobilization after surgery, expanded surgical expertize (external training visits for surgeons), closer collaboration with other departments about postoperative care and follow-up. The collaborations were tested and developed over time. | Complicated | Clear | Required learning | Staff and managers | Adaptive and technical | **Analyze**  Respond  **Categorize**  **Probe**  Sense  Respond |
| **37** | **5 GYN**  Cervical Cancer | Complicated situation – with a clear problem definition: Too long admissions due to urine retention and a one-size fits all follow up. The solution was developed through pathway mapping and a combination of the use of the obstetrical walking nurse in the PH. That allowed urine retention to be handled in PH. Individual needs assessment | Complicated | Clear | Required learning | Staff and managers | Adaptive and technical | **Analyze**  **Probe**  Sense  Respond |
| **38** | **6 GYN**  Vulva cancer | Complicated situation with a clear problem definition: too long and sometimes unnecessary admissions. The response was developed through process mapping and establishing a palliation conference and making individual plans and make admission in patient hotel an option. | Complicated | Clear | Required learning | Staff supported by managers | Adaptive and technical | **Analyze**  Respond  **Probe**  Sense  Respond |
| **39** | **7 GYN** Advanced uro-gynecology | Complicated situation with a clear problem definition: A wish to avoid admission after surgery. The responses were developed through process mapping and an iterative implementation of an outpatient set-up that involved OR and the postoperative observation unit – clinicians largely drove the process. The solution involved changes in surgical procedure (nausea, pain), faster mobilization and establishing a day surgical unit in the OR. It was a long “prototyping” period with adaption – monitoring of admissions. | Complicated | Clear | Required learning | Staff supported by managers | Adaptive | **Analyze**  Respond  **Probe**  Sense  Respond |
| **40** | **8 GYN**  Benign ovarian cyst | Complicated situation: Too much waiting time- for diagnostics and surgery – unnecessary visits – too long admissions. The response was developed through pathway analysis. It included that patients could have performed MRI in regional hospitals, changes in referral guidelines, reducing specialization level when cancer was not found. Full preoperative preparation in first visit, more minimal invasive surgery and faster mobilization and discharge. | Complicated | Clear | Required learning | Staff supported by managers | Adaptive and technical | **Analyze**  Respond  **Probe**  Sense  Respond |
| **41** | **9 GYN**  Bleeding disorder | Complicated problem that led to unnecessary visits, too much waiting time for surgery – to long admission after surgery. The response was developed through pathway mapping and probing improvement suggestions. Responses included the establishment of a central regions referral unit, new visitation guidelines. Standard surgical notes implemented and after surgery clear discharge plan should be present, to ensure smooth discharge all day (24/7). Fixed days for surgery and ambulatory for benign gynaecologist were implemented. | Complicated | Clear | Required learning | Staff and managers | Adaptive and technical | **Analyze**  **Probe**  Sense  Respond |
| **42** | **10 GYN**  Endometrial cancer | Complex situation with a problem definition that required learning – to much waiting time, not achieving target for national bundles requirements. The response was developed through process mapping and included new referral guidelines, implementation of full individualized diagnostic and preoperative visit before and during the first visit. Diagnostic test (MRI, hysteroscopy) planned in the same day. Answer and plan from multidisciplinary conference could be given via telephone, blood compatibility testing done in morning of surgery day allowing to be admitted on day for surgery, more minimal invasive surgery (more robotic capacity) full postoperative plan written after surgery – reduced amount of follow-up visits at AUH | Complex | Requires learning | Required learning | Staff and managers | Adaptive | **Analyze**  Respond  **Probe**  Sense  Respond |
| **43** | **1 GYN multiple**  Need assessment | Simple situation with a clear problem definition: That national requirement of individual needs assessment for cancer patients should be implemented. Change was to respond and follow this new external guideline. | Simple | Clear | Clear | Staff and managers | Technical and adaptive | **Respond** |
| **44** | **2 GYN multiple**  Less gyn-cancer follow-up ambulatories /week.  Reduced from 9 to 5(part of expanding surgical capacity) | Simple situation – with a clear problem definition: to many follow-up visits that specialist conducted instead of seeing newly referred bundle-care patients. Yet this prioritization was not something everybody agreed on. The response was developed and matured in an iterative process, however consensus was difficult to achieve – and changes was finally driven from top-management | Simple | Clear | Required learning | Managers | Technical and adaptive | Sense  **Categorize**  **Probe**  Sense  Respond  (top-down) |
| **45** | **3 GYN multiple**  Patient meet in the same day | Complicated situation with a clear problem definition – to many preoperative visits and a prolonged LOS due to admission the day before surgery. The response was developed through a review of pathways and made possible through new GOC settings – better coordination and collaboration that allowed patient to become fully preoperative ready in the first visit, much preoperative info can be handled via phone, blood compatibility test on the same day. | Complex | clear | Required learning | Staff and managers | Adaptive | **Analyze**  Respond  **Probe**  Sense  Respond |
| **46** | **4 GYN multiple**  Weekly palliation conference | Complicated situation with clear problem definition. Too long LOS for palliative patients.  The response was developed through analyzing pathways of palliative patient on the initial gyn camp. | Complicated | Clear | Required learning | Staff with support from managers | (Adaptive ) and technical | **Analyze**  Respond |
| **47** | **5 GYN multiple**  Early mobilization after surgery | Complicated problem – to long admissions after surgery.  Decision process: Pathways were analyzed and a pattern emerged that faster mobilization could make discharge after surgery faster complicated. The solutions included the use of chewing gum, faster catheter removal. Faster physical mobilization and pain control. | Complicated | Clear | Required learning | Staff supported by managers | Adaptive | **Analyze**  **Probe**  Sense  Respond |
| **48** | **6 GYN multiple**  More minimal invasive surgery | Complex situation with a problem definition that required learning. As surgical technique developed that potential to reduce LOS by performing more laparoscopic surgery was used. This approach was used before the downsizing requirements – however the task of reducing beds could potentially had a synergistic effect which further reduced LOS. The response was to aim for all minimal invasive procedures to be performed as day-surgery, which was a pattern that emerged in the first camp. In analysis of many pathways this component was seen as a possible response, however the full potential was not systematically recognized in the initial analytical work. | Complex | Required learning | Required learning | Staff supported by managers | Adaptive | **Analyze**  Respond  **Probe** |
| **49** | **1 GYN multiple**  Referral (external and internal) flow in the department:  Changed visitation | Complex situation with solutions that required learning. There were unnecessary visits, cancer-bundles where target was not archived. To long waiting list in AUH- capacity in regional departments. The solution was developed through mapping pathways and was largely influenced by an “external” requirement to establish a regional referral unit and that the outpatient clinic should move to a new location in the department.  Central referral unit, changed referral guidelines for cancer patients, no comfort scan in early pregnancy, emergency patient can be referred to other hospital if overbooked department and changing working hours for nurses.   1. Establish a regional central referral unit 2. Establishing extra ambulatories for bundle patients 3. Regional agreement that emergency patient can be referred to other hospital in case of a full department 4. Changed working hours for a nurse | Complex | Required learning | Required learning | Managers (and staff) | Adaptive and technical | **Analyze**  Respond  **Probe**  Sense  **Categorize** |
| **40** | **2 GYN org**  Changes in physical space  New gyn ambulatory setting. | Complex situation, with a response that required learning. Too much waiting time and unnecessary visits – lack of diagnostic capacity caused suboptimal care. The response was developed though mapping pathways combined with an external requirement to move location of the OGC where all subspecialties can be located in the same unit   1. Establish a new gynecological ambulatory with all sub-specialties in the same physical space – and available anesthesiology support. 2. Establish a coordinator nurse function for cancer bundles in the ambulatory. 3. Establish secretary teams located in ambulatory settings   Changes were made in scheduling which were probed and adapted over time. | Complex | Required learning | Required learning | Staff and managers | Adaptive and technical | **Analyze**  Respond  **Probe**  Sense  Respond |
| **51** | **3 GYN org**  Flow and capacity | Complex situation, with responses that required learning to develop. There was too much waiting time for surgery = unable to reach national bundle requirements. – many elements were included in responses, which developed over time in an iterative process where managers and staff rep. tested ideas, discussed effects and came up with new responses that included a weekly planning meeting and reallocation of staff resources away from follow-up ambulatories (9->5) towards more OR-lines, extra sub-acute ambulatories and shared OR-line between different subspecialist teams. Gyn. senior resident should supervise juniors in DSU (liberates specialists), more robotic surgery capacity and an expanded collaboration with abd. surgery was established. Surgeons, was developed in collaboration between DSU, OR, ward and subspecialized teams   1. Changed physician staffing in nightshift, which increased the amount of specialist during the day shifts. 2. Expanded surgical capacity, more robotic surgery, more surgical lines and training of surgeons. 3. Establish a weekly planning meeting to ensure optimal use of ambulatories and operating theater. | Complex | Required learning | Required learning | Staff and managers | Adaptive and technical | **Analyze**  Respond  **Probe**  Sense  Respond |
| **52** | **4 GYN org**  Better utilization of beds and faster discharge and Incl. a new nurse coordinator | Complex situation with a problem definition that required learning – LOS was too long and there were unnecessary admissions. The response (was developed through process mapping of all pathways and collaboration with other departments over time.  The responses included expanded collaboration with regional departments, a regional contact person for each specific sub-specialty, (arrangements for moving patients home or to regional departments after highly specialized care is ended), ward rounds in the morning to make plans and discharge smoothly, move multi-disciplinary meeting to utilize bed-use, GP- physician sweeper-function, faster mobilization after surgery, advanced uro-gynecology as day-surgery, shared understanding of early discharge, involve relatives, ascites drainage in ward instead of OR, bleeding after conus handled in EGC, weekly palliation conference.   1. Formalized agreements with other department and municipality about transfer of patients 2. New ward round routines to ensure smooth discharge 3. Establish a physician (GP-resident) support function to do ward round. 4. Establish a culture that facilitates discharge 24/7 5. Weekly palliation conference | Complex | Required learning | Required learning | Staff and managers | Adaptive and technical | **Analyze**  Respond  **Probe**  Sense  Respond |
| **53** | **1 org both OB&GYN**  Quality and safety nurse | Simple problem with a clear problem definition. | Simple | Clear | Clear | Managers | Technical | Sense  Respond |
