# Supplementary material for: Make it complicated: a qualitative study utilizing a complexity framework to explain improvement in health care
Source: BMC Health Serv Res. 2019 Nov 14;19:842. doi: 10.1186/s12913-019-4705-x (PMC6857274; doi:10.1186/s12913-019-4705-x)
Supplement: Supplementary file 2 — Additional file 2. Table of organizational situations addressed. [file 12913_2019_4705_MOESM2_ESM.docx]

# Additional File 2

Table of organizational situation addressed

| **Organizational Situation** | **Changes in Obstetrics** | **Changes in Gynecology** |
| --- | --- | --- |
| Referral (external and internal) and flow into department | 1. Expanded function of nurse coordinator in Obstetrical Outpatient Clinic 2. Establish a coordinator midwife function in the Midwife Clinic 3. Partus telephone | 1. Establish a regional central referral unit 2. Establishing extra ambulatories for cancer bundle patients 3. Regional agreement that emergency patient can be referred to other hospital in case of a full department |
| Changes in physical space | 1. Establish new obstetrical settings with 4 units (After Birth Clinic, Emergency Obstetrical Clinic, Pregnancy Ward and Labor-ward) in the same physical space. | 1. Establish a new gynecological ambulatory with all sub-specialties in the same physical space – and available anesthesiology support. 2. Establish secretary teams located in ambulatory settings |
| Flow and capacity | 1. Changed staffing, task shifting and more collaboration among nurses and midwifes | 1. Changed physician staffing in nightshift, which increased the amount of specialist during the day shifts. 2. Changed working hours for a nurse 3. Increased surgical capacity, more robotic surgery, more surgical OR-lines and training of surgeons. 4. Establish a weekly planning meeting to ensure optimal use of ambulatories and operating theater. 5. Establish a coordinator nurse function for cancer bundles in the ambulatory |
| Faster discharge after admission | 1. Establish a “walking nurse” function to care for patient in Patient Hotel and After Birth Clinic 2. Formalized agreements with other department and municipality about transfer of patients. 3. Establishing an After-birth Clinic with a 24/7 access telephone hotline to handle patient that was discharged faster. 4. Establish “home-team” for vulnerable families 5. Establish a culture that facili­tates discharge 24/7 | 1. Formalized agreements with other depart­ment and municipality about transfer of patients 2. New ward round routines to ensure smooth discharge 3. Establish a physician (General Practi­tioner-resident) support function to do ward round. 4. Establish a culture that facilitates discharge 24/7 5. Weekly palliation conference |
| Managerial support | 1. Employ a Quality and Safety nurse in the department to support management (OB & GYN) | |
